# Opposing Effects of Plant Invasion on the Stability of Aboveground and Belowground Net Primary Productivity in an Alpine Grassland

**DOI:** 10.1002/ece3.71730

**Published:** 2025-07-14

**Authors:** Qiu‐Jie Ren, Kai‐Hui Li, Heng‐Fang Wang, Yan‐Yan Liu, Yan‐Ming Gong

**Affiliations:** ^1^ College of Ecology and Environment Xinjiang University Urumqi China; ^2^ Xinjiang Institute of Ecology and Geography Chinese Academy of Sciences Urumqi China; ^3^ Bayinbuluk Alpine Grassland Observation and Research Station of Xinjiang, Xinjiang Institute of Ecology and Geography Chinese Academy of Sciences (CAS) Bayinbuluk China; ^4^ Key Laboratory of Oasis Ecology of Ministry of Education Xinjiang University Urumqi China

**Keywords:** biodiversity, ecosystem functioning, *Pedicularis kansuensis* invasion, species asynchrony, species richness

## Abstract

Plant invasion significantly disrupts plant community structure and ecosystem functioning, especially the stability of net primary productivity (NPP). However, evidence remains scarce regarding how invasion affects NPP stability at both community and ecosystem levels, particularly whether these effects are consistent between aboveground and belowground systems. Here, we investigated the responses of the stability of both aboveground and belowground NPP (ANPP and BNPP) to the invasion of the parasitic plant *Pedicularis kansuensis*, based on a two‐year manipulative experiment in an alpine grassland in northwest China. Invasion decreased ANPP resistance while increasing its recovery and conversely increased BNPP resistance but decreased its recovery. Notably, the asymmetric responses of ANPP and BNPP to invasion underscored the complexity of grassland ecosystems and highlighted the critical role of belowground processes in maintaining ecosystem recovery. Species asynchrony and richness were key factors for ANPP stability, whereas BNPP stability was more directly influenced by invasive species, contributing more significantly to overall NPP stability. Our findings provide crucial evidence that the stability of aboveground and belowground components responds asymmetrically to invasion, emphasizing the need for future comprehensive assessments of both dimensions in ecosystem studies. The insights gained underline the importance of belowground stability for sustaining ecosystem recovery and offer guidance for ecological management and conservation strategies.

## Graphical abstract

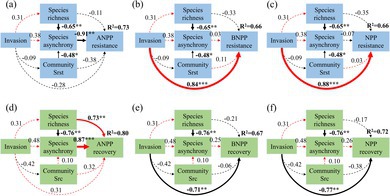

## 1 Introduction

Plant invasions significantly disrupt plant community structure and ecosystem functioning, particularly by compromising the stability of net primary productivity (NPP)—a fundamental ecosystem process that regulates energy flow, carbon sequestration, and overall ecosystem recovery (Ma et al. 2024). The impact of invasive plants on ecosystem stability has become increasingly significant, particularly in alpine grasslands, which are highly sensitive to global climate change. As one of the most sensitive ecosystems to climate change, alpine grasslands are experiencing dramatic environmental changes, such as rising temperatures and altered precipitation patterns. These changes may facilitate more hospitable living spaces for invasive plants, exacerbating their impact on local ecosystems (Chiu et al. 2023). Climate change, as a major driver of global change, has facilitated the spread of invasive plants (Liu et al. 2017), leading to substantial alterations in community structure and ecosystem functions (Eldridge and Ding 2021). While extensive research has focused on the effects of climate change on ecosystem stability, the impact of invasive plants on the stability of belowground NPP (BNPP) remains relatively little studied (Ma et al. 2023; Xu et al. 2024; Yang et al. 2022). As the frequency and intensity of droughts and other extreme climate events have increased substantially globally due to human activities, it has become particularly urgent to explore the factors affecting ecosystem stability. These changes not only disrupt the material cycling and energy flow within ecosystems but also exert profound impacts on biodiversity and ecosystem services (Ma et al. 2017; Wu et al. 2024).

The stability of ecosystems is pivotal in addressing global change, and research on this issue is of paramount importance. As a fundamental ecosystem characteristic, ecosystem stability refers to the capacity of an ecosystem to maintain relatively stable inter‐annual primary productivity under varying environmental conditions (Ma et al. 2017). This trait reflects how ecosystems respond to persistent and unpredictable disturbances, which is critical to the ecosystem functions and services that humans rely on (Zuo et al. 2023). Current research primarily focuses on the effects of climate change on ecosystem stability, particularly how temperature increases and changes in precipitation patterns affect ecosystem structure and function. Furthermore, the impacts of natural disturbances (e.g., drought) and anthropogenic disturbances (e.g., nitrogen enrichment, grazing, and fencing) on ecosystem stability have also garnered widespread attention (Carlsson et al. 2017; Song et al. 2023; Wang et al. 2023; Xu et al. 2022; Zhang, Chen, et al. 2024).

Biomass production, particularly NPP, is a fundamental indicator of ecosystem function (He et al. 2022; Sun et al. 2022), influencing energy flow, carbon sequestration (Yang, Stevens, et al. 2023), and resource allocation (Qiao et al. 2023). Both ANPP and BNPP are critical for assessing ecological stability, as they mediate key processes such as nutrient cycling, soil organic matter formation, and water retention (Schuur et al. 2001). While ANPP is often used as a proxy for ecosystem productivity, BNPP typically constitutes a larger fraction of total NPP, particularly in alpine grasslands, where root systems are essential for resource acquisition under harsh conditions (Kuhn et al. 2022). Belowground biomass dynamics are highly sensitive to environmental fluctuations, as root growth responds directly to water and nutrient availability, making BNPP a crucial yet understudied component of ecosystem resilience (Maan et al. 2023). Photosynthetically derived carbohydrates not only fuel root development but also regulate root architecture and foraging strategies, linking ANPP and BNPP in a feedback loop that shapes overall plant productivity (Zhang, Zhou, et al. 2023). Although drivers of ANPP stability—such as climate variability and species interactions—may similarly influence BNPP (Zhao et al. 2024) research on belowground stability remains limited, partly due to methodological challenges in quantifying root dynamics. Overcoming these limitations is essential for unraveling the mechanisms governing BNPP stability and its role in maintaining ecosystem functions, particularly in nutrient‐poor or water‐limited systems (Ma et al. 2024; Yang, Yang, et al. 2023).

Research indicates that invasive species can significantly affect both ANPP and BNPP (Wu et al. 2018). For instance, invasive plants often outcompete native species, leading to changes in community composition and structure (Rezacova et al. 2021). Plant functional group diversity plays a crucial role in resisting invasions, with studies showing that functionally similar species interact more intensely and exhibit stronger resistance to each other compared to dissimilar species (Dukes 2001; Mason et al. 2009). Different functional groups respond variably to invasions, with some functional groups experiencing substantial declines, relative stability, or even increases in ANPP (Pokorny et al. 2005). This variability in response is often linked to the competitive strategies of invasive species, their ability to exploit resources, and their interactions with native species (Mason et al. 2009). Additionally, invasions can alter soil properties (Schrama and Bardgett 2016), nutrient cycling (Sun et al. 2022), and microbial communities (Zhang et al. 2020). Some invasive plants may enhance the mutualistic plant–soil relationship through root exudates, promoting root growth (Tian et al. 2021; Yu et al. 2022). These changes can indirectly influence root development and BNPP, thereby affecting ecosystem productivity and stability. However, while the effects of invasive species on ANPP have been widely studied, BNPP remains relatively understudied, despite its critical role in ecosystem functioning. In summary, while the effects of nitrogen enrichment, temperature increases, and changes in precipitation patterns on ecosystem stability are well documented, the role of global change as a critical driver of invasions has only recently gained attention.

When discussing ecosystem stability, it is important to consider multiple dimensions. Ecosystem stability includes multiple dimensions, such as stability, resistance, and recovery. Some experimental studies have determined that functional recovery is positively connected to community structural recovery (Mitchell et al. 2006). Therefore, community structure stability is also an important factor in predicting the functional stability of ecosystems in response to global change (Ma et al. 2024). Each dimension responds to the environmental changes experienced by the ecosystem, and the relationships between the stability of different dimensions differ (Zhang and Wang 2023). However, most research on ecological stability has focused on a single dimension of stability, which has the potential to bias results concerning the overall stability of ecosystems. The adoption of multidimensional frameworks in stability studies is increasingly important for a comprehensive understanding of ecological stability and effectively characterizing ecosystem dynamics in response to global environmental change (Ma et al. 2024).

With increasing research on the impact of ecosystem productivity, species diversity is recognized as a key determinant of ecosystem productivity stability. The diversity–stability hypothesis posits that higher species diversity leads to more stable ecosystems (Craven et al. 2018). This is because diversity enhances the resilience and recovery of ecosystems, enabling them to better withstand environmental changes and disturbances. In ecosystems, species richness and species asynchrony are the core of responding to environmental change and are important driving forces for the functional stability of ecosystems (Yan et al. 2023; Zhang, Bai, et al. 2023). The emergence of invasive plant species may disrupt this diversity–stability relationship by altering interspecies interactions, thereby reducing the stability of the ecosystem (Vetter et al. 2020). In the context of environmental change, high species richness and heterogeneity can promote ecosystem diversity and stability. For instance, ecosystems with high species richness and good heterogeneity may exhibit greater recovery and productivity under climate change, drought, or other disruptive events. This mechanism provides crucial insights into how ecosystems adapt and respond to change. Previous studies demonstrated that invasive alien species often disrupt the balance of local species (Beaury et al. 2023; Guido and Pillar 2017), leading to a decline in ecosystem stability. Current research highlights that biological invasions affect ecosystem stability through multiple mechanisms. For example, habitats experiencing disturbance patterns (such as the frequency and intensity of disturbances) that differ from historical patterns tend to be more vulnerable to invasion by alien species (Jauni et al. 2015). Biotic resistance varies, with some alien species inhibited in competition with native species, while others have a competitive advantage (McGlone et al. 2011). The effects of different invasive plant species on ecosystems are diverse (Zeil‐Rolfe et al. 2024). On one hand, some exotic plants can successfully adapt to the new environment and replace native plants due to high reproduction and diffusion rates, leading to a decline in ecosystem diversity (Oduor 2022). On the other hand, some exotic plants may form mutually beneficial relationships with native plants, enhancing certain ecological processes (Mitchell et al. 2006).

Alpine grasslands, however, present a distinct scenario where invasion impacts may deviate from conventional expectations due to their stringent abiotic filters and tightly coupled species interactions. Alpine grassland is a unique ecosystem distributed in high‐altitude areas, serving as an ideal model system for studying invasion ecology due to its heightened sensitivity to environmental changes (Li et al. 2019). Plants in this environment face harsh living conditions, including short growing seasons, high UV radiation, low temperatures, nutrient availability, and soil microbial activity (Duan et al. 2021; Sun et al. 2024). These environmental pressures have created special plant community structures and physiological adaptation mechanisms, making the plant community of alpine meadows highly ecologically sensitive. The interspecies competition and coexistence of alpine meadow ecosystems are affected by their unique environmental conditions. Due to the scarcity of resources, competition between plant species is often extremely fierce, and this environmental pressure also promotes symbiotic mechanisms among plants (e.g., use of symbiotic bacteria and rhizosphere microorganisms) (Jiang et al. 2018), rendering alpine meadows particularly vulnerable to biological invasions under climate warming scenarios. The invasion of *Pedicularis kansuensis*, a root hemiparasitic plant, may influence ecosystem stability through mechanisms that differ from typical invasive species. Unlike conventional invaders that primarily compete for resources directly, *P. kansuensis* appears to establish more complex trophic interactions through its semi‐parasitic relationship with host grasses, potentially including selective parasitism of dominant species and possible modulation of belowground microbial networks. Over the past two decades, *P. kansuensis* has extensively colonized the Bayinbuluk alpine grassland, leveraging its high adaptive and reproductive capacity to assume a dominant ecological role. This invasion provides a critical opportunity to examine how parasitic traits, particularly host dependence and hemiparasitism, influence ecosystem resilience in extremely high‐altitude environments. Under the dual pressures of global climate change and biological invasions, alpine grassland ecosystems are facing unprecedented challenges. Consequently, studying the impacts of invasions on ecosystem stability has become particularly crucial. Here, using an experiment with *P. kansuensis* removal manipulation over 2 years in an alpine grassland in northwest China, we aimed to explore (1) how invasions influence multiple dimensions of ecosystem function and community structure stability (e.g., resistance and recovery), particularly the understudied impacts on BNPP in alpine grasslands, (2) clarify the relationship between ecosystem functional stability and community structural stability, and (3) elucidate the dominant pathways through which invading influences ecosystem functional stability.

## 2 Materials and Methods

### 2.1 Study Site

Samples of *P. kansuensis* and co‐occurring native species were investigated in the Bayinbuluk alpine grassland, Xinjiang Province, China (42°53′08″ N, 83°42′27″ E, elevation 2460 m a.s.l.). The sampling location has a temperate continental dry climate, with an average annual temperature of −4.8°C and average annual precipitation of 300.8 mm (Liu et al. 2022). The climate is characterized by large seasonal temperature fluctuations, with cold winters and short, cool summers. The soil is classified as Cambisol according to the Food and Agriculture Organization soil classification system, with high organic matter and nitrogen content but relatively low phosphorus levels (Liu et al. 2022). The selected plant community is herbaceous (without trees or shrubs), predominantly composed of Gramineae (Poaceae) species such as *Festuca kryloviana* and *Stipa purpurea* as the dominant species, and *P. kansuensis* as the sole invasive plant in the invaded communities. The study was conducted in alpine grassland habitats, characterized by a high degree of plant diversity, with over 20 species recorded in the study area.

### 2.2 Experimental Design and Sampling

Within a 100 m × 100 m large plot, five 10 m × 10 m plots were randomly selected. In each plot, three subplots with *P. kansuensis* invasion (invasion—affecting the host under natural conditions), three subplots without *P. kansuensis* (non‐invasion—no effect on the host), and three subplots with artificial removal of *P. kansuensis* (remove *P. kansuensis* —already affecting the host during the seedling stage) were randomly selected. Each subplot measured 1 m × 1 m. From early June, *P. kansuensis* seedlings were manually removed every 3 days for 1 month to ensure the absence of *P. kansuensis* within the ‘remove *P. kansuensis* ’ plots. Sampling was conducted over two consecutive years (2014–2015), with plots remaining fixed and subplots being randomly assigned each year. Plant community surveys were carried out in mid‐August, during the peak biomass period of the plants.

Plant cover was assessed by positioning a 1 × 1 m frame containing 100 grids of 10 × 10 cm. Within each quadrat, the majority of plants were identified to the species level in the field, and the percent cover of each species was estimated based on their occurrence within the 100 grids. Percent coverage was summed across species to obtain total community cover. Species richness was quantified as the number of species recorded within each quadrat (Xu et al. 2022).

For ANPP measurements, a 50 cm × 50 cm frame was placed within each quadrat. All plant material within the frame was clipped at ground level, cleaned of soil and gravel, and taken to the laboratory. Samples were dried at 65°C until constant weight was achieved, and ANPP was determined by weighing using a balance for each species. The classification information of plant species, families, and genera, along with the categorization of plant functional groups (grasses, forbs, and legumes) in the sample subplots, is detailed in Table S1.

For BNPP measurements, we employed a root ingrowth‐core method to quantify BNPP in our perennial‐dominated study system (Liu et al. 2018; Ma et al. 2017). In September 2013, we extracted three soil cores (8 cm diameter × 30 cm depth) per plot, sieved the soil through 2 mm mesh to remove existing roots, and refilled the holes with root‐free soil contained within 1 mm mesh bags. During subsequent growing seasons (2014–2015), we collected the ingrowth cores at ANPP sampling dates, separating roots by depth interval (0–10, 10–20, 20–30 cm) (Liu et al. 2018; Ma et al. 2017; Wang et al. 2023). Visible organic matter (including leaf litter > 2 mm) and lithic particles (gravel > 2 mm) were manually removed from field‐moist soil samples using forceps. The samples underwent air‐drying in a climate‐controlled laboratory environment (18°C ± 2°C, relative humidity 50%–60%) for 48 h to achieve constant weight. Dried samples were sieved through a 2‐mm aperture mesh to achieve: retention of coarse fragments and root materials (> 2 mm); collection of the fine earth fraction (< 2 mm) for subsequent analysis. The sieved roots were weighed (BNPP—belowground biomass), and the remaining soil was used for physicochemical property measurements. The NPP represents the sum of ANPP and BNPP. Soil organic carbon content (SOC) was measured by wet oxidation using the Walkley‐Black method (Hardy and Dufey 2017), which involves digesting soil with a mixture of potassium dichromate and sulfuric acid to quantify oxidizable organic matter. Soil total nitrogen (SN) was analyzed via the Kjeldahl method (Allende‐Montalbán et al. 2024) using a Kjeltec System 2300 Analyzer, where soil is digested with sulfuric acid to convert organic nitrogen into ammonium, followed by distillation and titration. Soil total phosphorus (SP) was determined by colorimetry after digestion with HClO_4_‐H_2_SO_4_ (John 1970): the released phosphate reacts with molybdate and ascorbic acid to form a blue complex, measured spectrophotometrically. Soil total potassium (SK) was quantified by inductively coupled plasma optical emission spectrometry (ICP‐OES) after acid digestion, which atomizes elements and detects their emission spectra. Soil pH was measured in a 1:5 (w/v) soil‐water suspension using a calibrated pH meter (Bao 2000).

### 2.3 Calculations of Ecological Stability

We quantified the resistance and recovery of both biomass and community composition as measures of functional and structural stability (van Ruijven and Berendse 2010; Chen et al. 2023). The NPP resistance (Brst) and recovery (Brc) were calculated using the following equations:

(1) $$\text{Brst}=\frac{{\mathrm{NPP}}_{\text{invasion}}}{{\mathrm{NPP}}_{\text{non}-\text{invasion}}}$$

(2) $$\mathrm{Brc}=\frac{{\mathrm{NPP}}_{\text{remove}\;P.K}}{{\mathrm{NPP}}_{\text{invasion}}}$$

where NPP_invasion_ is the NPP of invasion, NPP_non‐invasion_ is the NPP of non‐invasion, and NPP_remove P.K_ is the NPP with artificial removal of *P. kansuensis* .

A result of Brst = 1 indicates complete resistance, Brst = 0 indicates no resistance (no biomass is produced at the time of invasion), and Brst > 1 indicates an increase in NPP. A result of Brc = 1 indicates complete recovery, Brc < 1 indicates incomplete recovery, and Brc > 1 indicates overcompensation. The same method was used to calculate the resistance and recovery of ANPP and BNPP.

Community structural resistance (Srst) and recovery (Src) were calculated as the Bray–Curtis similarity between non‐invasive and invasive communities and the Bray–Curtis dissimilarity between removed *P. kansuensis* and invasive communities (Bray and Curtis 1957). The equations follow:

(3) $$\text{Srst}=1-\sum_{i}\left|{\mathrm{RC}}_{i,\text{non}-\text{invasion}}-{\mathrm{RC}}_{i,\text{invasion}}\right|/2$$

(4) $$\mathrm{Src}=1-\sum_{i}\left|{\mathrm{RC}}_{i,\text{remove}\;P.K.}-{\mathrm{RC}}_{i,\text{invasion}}\right|/2$$

where RC_i,non‐invasion_, RC_i,invasion_, and RC_i,remove P.K_ are the relative coverage of the *i*th species in the non‐invasive, invasive, and remove *P. kansuensis* communities, respectively. A result of Srst = 1 indicates no structural difference between invasive and non‐invasive communities, and Srst = 0 indicates no common species between the invasive and removed *P. kansuensis* communities. The maximum value of Src ≈ 1 indicates that ANPP is significantly greater after than before invasion; however, Src < indicates low recovery.

Species asynchrony in each plot every year was quantified as species coverage (the coverage of species emerging in the frame of the subplot). Species asynchrony was calculated (Loreau and de Mazancourt 2008):

(5) $$\varphi_{y}=1-\varphi_{x}=1-\frac{\sigma^{2}}{{\left(\sum_{i=1}^{n}\sigma_{i}\right)}^{2}}$$

where *φ*_*y*_ and *φ*_*x*_ are species asynchrony and synchrony for each subplot, respectively; *σ* ^2^ is the variance of plant community ANPP; and *σ*_*i*_ is the standard deviation of the ANPP of the *i*th species in a subplot with n species.

For the calculation of Srst and Src, species asynchrony, and linear fitting, we utilized plant coverage, rather than biomass, as a proxy for primary productivity (Xu et al. 2022). Within grassland ecosystems, non‐destructive plant coverage surveys circumvent the disturbances associated with biomass estimation, and there is a robust positive correlation between plant species coverage and biomass (Sanaei et al. 2018; Xu et al. 2022).

### 2.4 Statistical Analysis

One‐way analysis of variance (ANOVA) was conducted to test the independent effects of invasion on multiple dimensions of ANPP, BNPP, and NPP stabilities and plant community properties (i.e., species richness, species asynchrony, and stability of community structure). Prior to ANOVA, we verified that: (1) Normality was tested using Shapiro–Wilk tests (*p* > 0.05); (2) Homogeneity of variance was assessed via Levene's test (*p* > 0.05). If assumptions were violated, we applied data transformations (e.g., log‐transformation) or used non‐parametric alternatives (Kruskal‐Wallis test). The differences were tested using Tukey's honestly significant difference (HSD) method. Simple linear regressions were applied to assess how multiple stability dimensions (i.e., community structure stability and ecosystem functioning stability) and plant community properties varied with invasion levels. Finally, we used structural equation modeling (SEM) to examine how *P. kansuensis* invasion influenced multiple stability dimensions through its effects on species richness, species asynchrony, and community structure stability. The SEM was constructed based on hypothesized causal pathways derived from ecological theory and prior studies. We first developed a conceptual model specifying direct and indirect relationships between invasion status (presence/absence or abundance of *P. kansuensis* ), biodiversity components (richness, asynchrony), and stability metrics (resistance, recovery). Model fit was evaluated using multiple criteria: *χ* ^2^ test (non‐significant *p*‐value indicates good fit). Akaike Information Criterion (AIC) for model comparison. Root Mean Square Error of Approximation (RMSEA) (values < 0.05 suggest close fit; < 0.08 acceptable). Analyses were performed in AMOS 22.0 (Amos Development Corporation), with data preparation and visualization conducted in RStudio 2024.04.0 (Posit Software) using ggplot2 (v3.4.0).

## 3 Results

### 3.1 Effects of Invasion on Ecosystem Functional Stability

**FIGURE 1:**
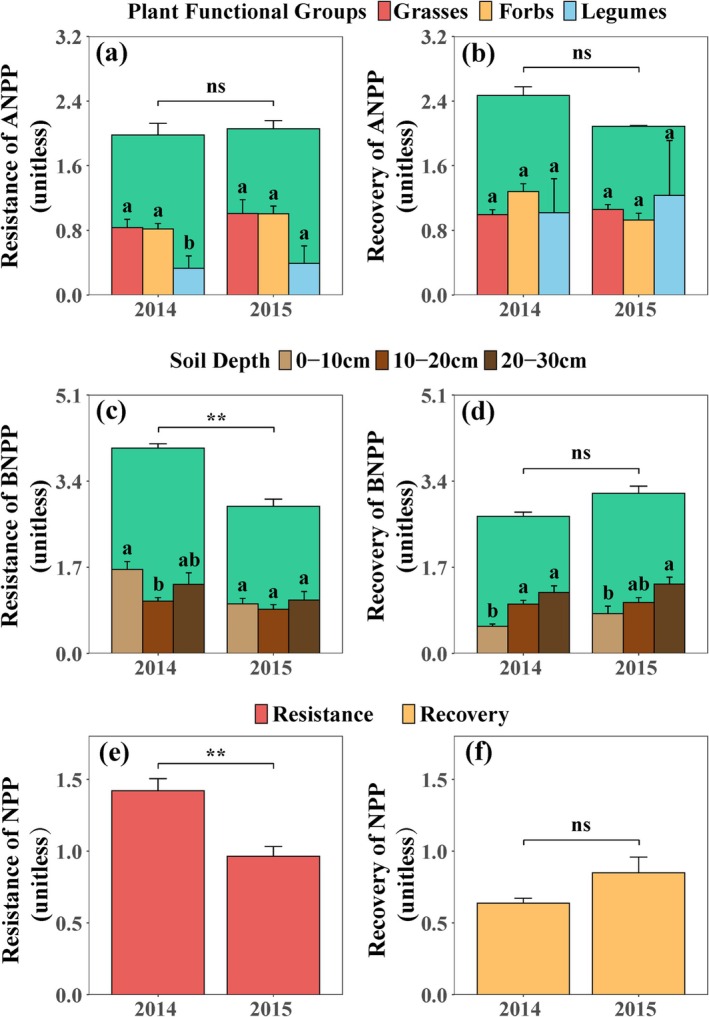
Effects of *P. kansuensis* invasion and removal of *P. kansuensis* on (a, b) aboveground net primary productivity (ANPP) resistance and recovery; (c, d) belowground net primary productivity (BNPP) resistance and recovery; and net primary productivity (NPP) (e) resistance and (f) recovery. Bars represent mean ± standard error (SE), *n* = 5. Note: **p < 0.01, ns = not significant (*p* ≥ 0.05). SE reflects the precision of the mean estimate, with smaller error bars indicating higher confidence in the treatment effects. Variability in the data may influence ecosystem stability indices (e.g., resistance or recovery to invasions). One‐way ANOVA was used for the difference comparison. Treatments sharing the same letter are not significantly different from each other in a post hoc Tukey's HSD test at *p* = 0.05. Aboveground parts of species were divided into three functional groups: grasses, forbs, and legumes. Belowground soil was divided into three depths: 0–10, 10–20, and 20–30 cm.

At the initial stage of invasion (in 2014), *P. kansuensis* significantly reduced the aboveground biomass of leguminous functional groups (*p* < 0.05, Figure 1a), but had no significant effect on grasses and forbs, consistent with the niche difference hypothesis (Lozon and MacIsaac 1997) where invaders preferentially exploit underutilized niches in specific functional groups. Invasion of *P. kansuensis* did not significantly affect the ANPP recovery of each plant functional group (Figure 1b), suggesting that aboveground disturbance effects may be transient or compensated by other processes. In contrast, belowground impacts were more pronounced: the invasion significantly decreased 10–20 cm BNPP resistance (*p* < 0.05, Figure 1c), supporting the disturbance hypothesis that invasive species create persistent belowground niche vacancies, thereby weakening native root system stability. The 0–30 cm BNPP resistance showed an overall significant decrease in the second year (*p* < 0.05, Figure 1c), indicating a cumulative negative effect on deeper root biomass over time. At the initial stage of invasion, *P. kansuensis* significantly reduced 0–10 cm BNPP recovery (*p* < 0.05, Figure 1d), and showed an overall increase from 0 to 30 cm in the second year (in 2015, Figure 1d). Invasion significantly decreased NPP resistance (*p* < 0.01, Figure 1e), and NPP recovery non‐significantly increased (Figure 1f).

### 3.2 Responses of Community Structure Stability to Invasion

**FIGURE 2:**
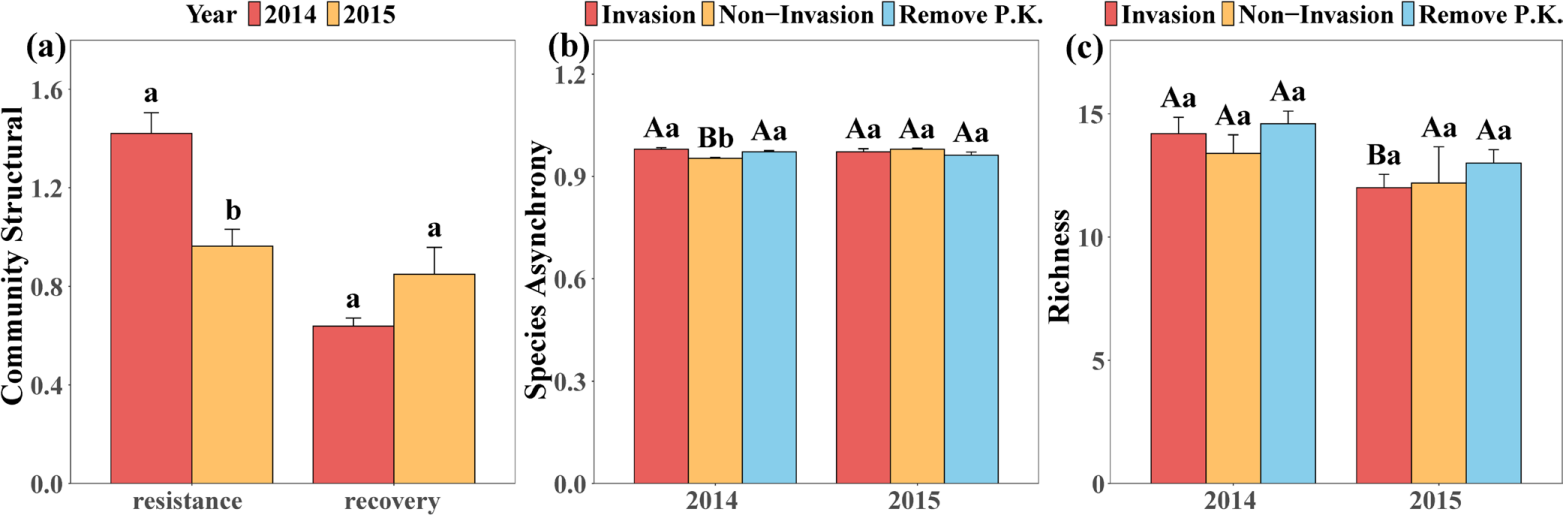
Effects of *P. kansuensis* invasion and removal on (a) community structural resistance and recovery, (b) species asynchrony, and (c) species richness. Bars represent mean ± SE, *n* = 5. SE reflects the precision of the mean estimate, with smaller error bars indicating higher confidence in the treatment effects. Data variability may influence the assessment of ecosystem stability indices (e.g., community structural stability, species asynchrony, and richness under invasion conditions). One‐way ANOVA was used for the difference comparison. Treatments sharing the same letter are not significantly different from each other in a post hoc Tukey's HSD test at *p* = 0.05. In Figures b and c, different capital letters indicate differences between years, and different lowercase letters indicate differences between groups.

After invasion, community structural resistance decreased significantly (*p* < 0.01, Figure 2a), and recovery of the community structure improved. At the beginning of the invasion, species asynchrony of the invasion and the artificial extraction groups was significantly higher than that of the non‐invasion group, and this pattern persisted in the second year (*p* < 0.05, Figure 2b). After 2 years of continuous invasion, species richness declined significantly. The non‐invasion and *P. kansuensis* removal treatment groups also experienced reduced species richness, although this was non‐significant (Figure 2c).

### 3.3 Relationships Between Structural and Functional Stability Dimensions

**FIGURE 3:**
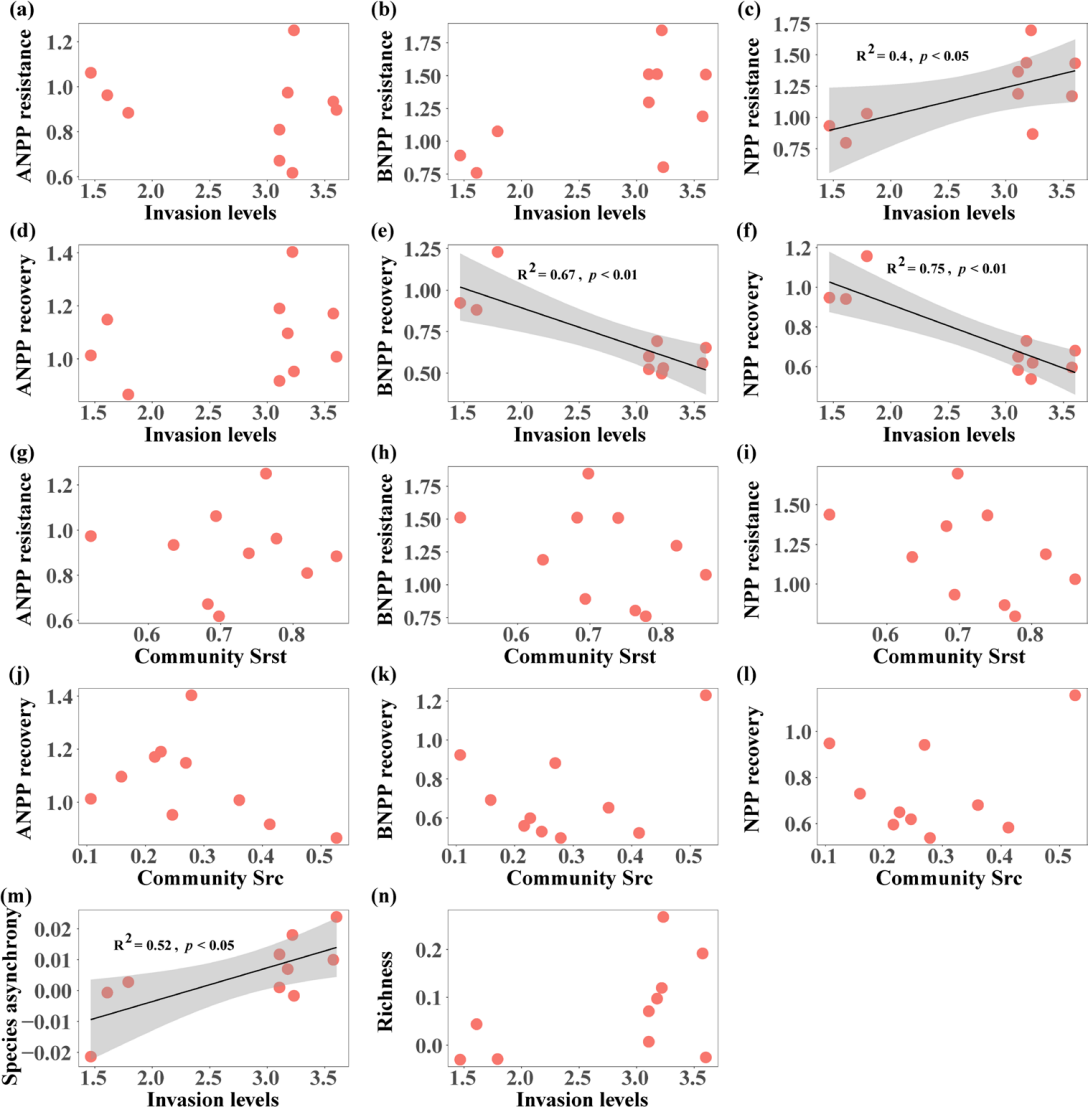
Relationships between compositional and functional stability dimensions across different invasion levels. ANPP, aboveground net primary productivity; BNPP, belowground net primary productivity; NPP, net primary productivity; Community Srst, community resistance; Community Src, community recovery. The level of *P. kansuensis* invasion is represented by its coverage. Black solid lines indicate significant regression lines (*p* < 0.05). Each orange solid point represents an experimental sample (*n* = 10). The shaded area denotes 95% confidence intervals.

A positive correlation was observed between NPP resistance (*p* < 0.05, Figure 3c) and species asynchrony (*p* < 0.05, Figure 3m) as the degree of *P. kansuensis* invasion increased. Conversely, with the increasing degree of *P. kansuensis* invasion, both BNPP recovery (*p* < 0.01, Figure 3e) and NPP recovery (*p* < 0.01, Figure 3f) gradually declined. There were no significant relationships between ecosystem functional stability and community structural stability (Figure 3g–i). Invasion had no effect on species richness (Figure 3n).

### 3.4 The Impacting Pathways of Invasion on Stability

**FIGURE 4:**
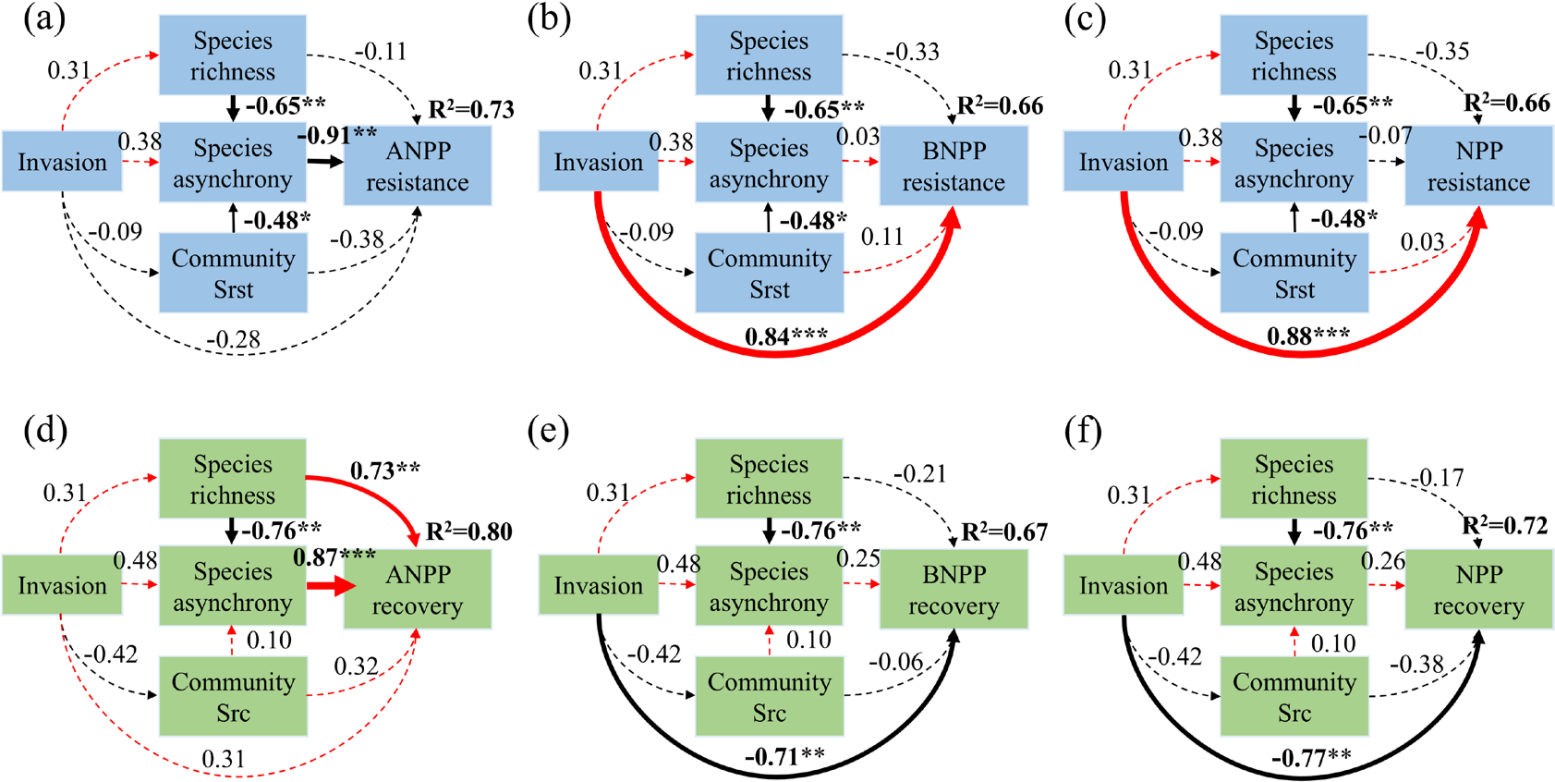
Final structural equation modeling showing direct and indirect effects of *P. kansuensis* invasion on (a) aboveground net primary productivity (ANPP) resistance and community structural resistance (Srst), (b) belowground net primary productivity (BNPP) resistance and community structural resistance (Srst), (c) net primary productivity (NPP) resistance and community structural resistance (Srst), (d) ANPP recovery and community structural recovery (Src), (e) BNPP recovery and community structural recovery (Src), and (f) NPP recovery and community structural recovery (Src). Red and black solid arrows indicate significant positive and negative pathways, respectively, and red and black dashed arrows represent non‐significant pathways; ***p < 0.001, **0.001 ≤ *p* < 0.01, and *0.01 ≤ *p* < 0.05. The *R* ^2^ values associated with variables suggest the proportion of variance explained by relationships with other variables. The values adjacent to arrows are standardized path coefficients. Arrow width is proportional to the strength of the relationship. Goodness‐of‐fit statistics: (a) *χ* ^2^ = 1.189, *p* = 0.275, df = 1, RMSEA = 0.145, AIC = 39.189; (b) *χ* ^2^ = 1.189, *p* = 0.275, df = 1, RMSEA = 0.145, AIC = 39.189; (c) *χ* ^2^ = 1.189, *p* = 0.275, df = 1, RMSEA = 0.145, AIC = 39.189; (d) *χ* ^2^ = 0.522, *p* = 0.470, df = 1, RMSEA < 0.001, AIC = 38.522; (e) *χ* ^2^ = 0.522, *p* = 0.470, df = 1, RMSEA < 0.001, AIC = 38.522; and (f) *χ* ^2^ = 0.522, *p* = 0.470, df = 1, RMSEA < 0.001, AIC = 38.522.

The SEM confirmed that invasion had an indirect effect on the stability of ANPP and a direct effect on the stability of BNPP and NPP. Species asynchrony is an important driver of ANPP stability. Species asynchrony, which was not altered by invasion, showed negative effects on ANPP resistance (Figure 4a). Species asynchrony and species richness, which were not altered by invasion (Figure 4d), showed negative effects on ANPP recovery. The SEM accounted for 73% of the variation in ANPP resistance to invasion and 80% of the variation in its recovery from invasion. Additionally, invasion had a direct positive impact on the resistance of both BNPP and NPP (Figure 4b,c); however, there were negative effects of invasion on the recovery of BNPP and NPP (Figure 4e,f). The explained variances of resistance for BNPP and NPP were both 66%, while the explained variances of recovery were 67% and 72%, respectively. In addition, most dimensions of ecosystem functional stability were largely independent of community structural stability with *P. kansuensis* invasion.

## 4 Discussion

Ecologists have long been captivated by the stability of natural ecosystems (De Mazancourt et al. 2013). Regrettably, many past plant‐centric studies—whether conceptual, model‐based, or experimental—have focused on the stability of plant ANPP (or its related indicators). In contrast, BNPP stability has been largely overlooked and little studied (Xu et al. 2024). Despite ongoing invasive behaviors and frequently reported severe damage to ecosystems caused by invasive plants, few studies have examined the effects of invasion on multidimensional ecosystem stability, and the impact of invasion on belowground stability has never been explored. We present the first evidence of the multidimensional stability of an ecosystem influenced by invasions. Interestingly, we discovered that the responses of aboveground and belowground stability to invasion were asymmetric. Specifically, invasion reduced ANPP resistance and increased ANPP recovery but increased BNPP resistance and decreased BNPP recovery. Invasive phenomena have a profound impact on grassland ecosystems, particularly in reshaping ANPP and BNPP stability (Bardgett et al. 2006; Hao et al. 2024). This asymmetric response of ANPP and BNPP stability to invasion fully illustrates the complexity of grassland ecosystems in the face of environmental disturbances. Therefore, to fully and deeply understand the response mechanisms of ecosystem stability, it is essential to consider both aboveground and belowground ecosystems from multiple dimensions (Mahaut et al. 2023; Polazzo and Rico 2021; Wilcox et al. 2017).

### 4.1 Responses of Grassland Ecosystem Functions to Invasions

Invasions caused by global change disrupt grassland productivity through asynchronous dynamics between species in response to natural environmental fluctuations. Resource‐conservative grasses determine biomass resistance and resource‐access grasses determine biomass recovery, suggesting that plant resource use strategies may play an important role in the trade‐off between drought resistance and recovery of grassland biomass (Mackie et al. 2019; Song et al. 2023). Compensation dynamics among species or functional groups have been extensively studied and are considered important mechanisms for maintaining functional stability of ecosystems (Connell and Ghedini 2015; Liu et al. 2018; Sasaki et al. 2019). In response to invasive species, the nitrogen‐fixing capacity of Fabaceae not only promotes invasion but also may be compromised to some extent, resulting in a decline in biomass (Byun et al. 2013; Mwangi et al. 2007), which may exacerbate the reduction of ANPP resistance in legume functional groups. In the second year of the experiment, the NPP recovery increased, indicating that in the face of the negative impact of environmental stress, the ecosystem ensured a relatively stable level of NPP by adjusting the physiological activity of functional groups and the complementarity between ecological niches, leading to more complete resource utilization and greater community resistance and stability (Gao et al. 2022; Lv et al. 2024; Qiao et al. 2023; Zhou et al. 2019). Grasses and forbs are the dominant functional groups, and their stability was not affected by invasion, which is an important factor affecting community stability in this experiment (Huang et al. 2020). All three plant functional groups were able to fully recover from invasion events and even overcompensate. When invasion events occur, parasitism may significantly impair the recovery of dominant plant functional groups, especially forbs. This effect may create favorable conditions for regeneration in other species, where grasses and legumes may show relative tolerance or even benefit from it. These species form complementary relationships with the affected forbs (Figure 1b), which together contribute to the restoration process of a highly diverse natural community (Tesitel et al. 2017).

We found that invasion had a positive impact on BNPP resistance and a negative impact on BNPP recovery, opposite to the impact of invasion on the stability of ANPP, indicating that aboveground and belowground ecosystems may exhibit different responses in the face of invasion. The enhanced BNPP resistance observed in this system likely stems from the specialized hemiparasitic ecology of *P. kansuensis* . As a root hemiparasite, *P. kansuensis* establishes direct haustorial connections with host root systems, facilitating efficient acquisition of both inorganic nutrients (particularly nitrogen and phosphorus) and organic carbon compounds (Ma et al. 2017; Tesitel et al. 2011). This unique nutritional strategy enables *P. kansuensis* to mitigate resource limitations during environmental stress periods, thereby contributing to BNPP stability. In contrast to the increased resistance of BNPP, the reduced recovery of BNPP indicates that the recovery process becomes extremely difficult when belowground ecosystems suffer damage, such as root destruction or soil structure changes. The cause of this phenomenon is that the *P. kansuensis* taproot mainly parasitizes in the 0–10 cm soil layer, which especially weakens the recovery of the ecosystem in this depth range. By contrast, species with deep roots are able to draw water and nutrients from deeper layers of soil and remain relatively stable in productivity in the face of invasion. This decline in BNPP recovery may, in the long term, adversely affect the stability and sustainability of the ecosystem.

### 4.2 Mechanisms Underlying Inconsistency in Aboveground and Belowground Ecosystem Stability

Consistent with the majority of research findings, we also corroborate that species asynchrony and species richness are critical determinants of aboveground functional stability (Sasaki et al. 2019; Schnabel et al. 2021). The insurance hypothesis states that species richness enhances the resistance and recovery of ecosystems and buffers the effects of disturbances on ecosystems (De Mazancourt et al. 2013; Hou et al. 2023). More diverse communities are more likely to include stress‐resistant species and the ability to recover quickly or compensate for other species (Hautier et al. 2014; Hou et al. 2023), increasing the likelihood that species in the community will respond differently to environmental conditions and disturbances (Cheng et al. 2024; Zhang et al. 2022). However, the research results on the impact of species richness on grassland ecosystems show a complex and changeable situation. Different species diversity gradients (Hossain et al. 2022; Zhao et al. 2024), differences in the intensity of climate events (Kreyling et al. 2017), and differences in management regimes (Vogel et al. 2012) can lead to different conclusions. Climate change may indirectly influence species richness and ecosystem productivity by affecting physiological activities and ecological niche complementarity (Han et al. 2023; Zhang, Gao, et al. 2024). Our meteorological data (Figure S1) revealed significant inter‐annual variations, with 2015 receiving 32% more annual precipitation (302.7 mm) than 2014 (229.0 mm), particularly evident in May (69.3 versus 20.4 mm) and August (70.2 versus 20.0 mm). Conversely, July 2014 received 2.5 times more precipitation (80.8 mm) than July 2015 (32.6 mm). Temperature differences were less pronounced, though 2015 was consistently warmer (annual mean −3.7°C versus −4.7°C), with July temperatures differing markedly (13.9°C in 2015 versus 10.6°C in 2014). These climatic asymmetries, especially the precipitation shifts between critical growing months (May–August), likely contributed to observed inter‐annual variations in species asynchrony and richness through differential effects on plant water availability and thermal stress tolerance. Furthermore, SEM further corroborates that species richness contributes to ANPP recovery (Figure 4d). The effect of species richness on ANPP resistance was not significant (Figure 4a), possibly due to the stronger influence of species asynchrony. This asynchrony promotes niche complementarity, allowing different species to utilize resources more diversely, thereby reducing competition and increasing overall community productivity. Species richness enhances community stability by providing a greater variety of ecological functions and resource use strategies, thereby increasing the community's recovery from environmental pressures.

Some studies have suggested that differences in plant functional group composition are the main reason for the decoupling of resistance and recovery of BNPP/NPP and ANPP (Carlsson et al. 2017; Gherardi and Sala 2020; Ma et al. 2024; Mackie et al. 2019). Previous studies have shown that the presence of *P. kansuensis* does not lead to significant declines in native species richness or significant changes in community structure (Liu et al. 2022). The current study further verifies this observation using SEM and clearly indicates that the stability of community structure is not a key factor affecting ecosystem stability. Our inspection showed that invasion did not change the soil physical and chemical properties (Figure S2). It is noteworthy that the inconsistent responses of aboveground and belowground stability to invasions may be due to the characteristics of parasitic species. The stability of NPP is largely regulated by BNPP and is not directly related to ANPP. The weakening of belowground stability may lead to a reduction in the stability of NPP, highlighting an impact on the overall ecosystem scale that has previously been overlooked because studies have focused on aboveground stability (Ma et al. 2024). Our study further underscores that an exclusive focus on aboveground stability is inadequate to elucidate the ecological ramifications of invasive behavior.

### 4.3 Limitations

Due to the lack of long‐term data, we employed the Space‐for‐Time Substitution (SFTS) method to simulate and predict the future changes of invasive species in the patterns of ecosystem change. (Kharouba and Williams 2024; Wogan and Wang 2018). That is, when the drivers of biological turnover in space coincide with those of biological turnover in time, space can be studied as an alternative fixed landscape for time. Although the SFTS method mainly relies on spatial validation due to the lack of long‐term data, it provides an effective research framework (Blois et al. 2013; Lovell et al. 2023). However, it is important to note that while our research has already provided valuable insights, extending the time frame of the study will further enrich our understanding of ecosystem stability. In addition, future studies may consider combining field and remote sensing data to build a more comprehensive and multidimensional ecosystem stability assessment system, which will help us more accurately predict and respond to possible challenges faced by ecosystems.

## 5 Conclusions

Our study provides insight into the impact of invasive species on multidimensional ecosystem stability. In particular, we found asymmetric responses of aboveground and belowground ecosystems in the face of invasion, fully demonstrating the complexity of grassland ecosystems in the face of environmental disturbances. The characteristics of invasive species are a key factor in this asymmetric response. In addition, our research highlights the importance of belowground ecosystem stability for overall ecosystem stability and sustainability. Future research should continue to explore the mechanisms underlying the stability of underground ecosystems and how invasive species affect these mechanisms to more fully understand the dynamics of ecosystem stability.

## Author Contributions

**Qiu‐Jie Ren:** conceptualization (equal), data curation (equal), formal analysis (equal), methodology (equal), software (equal), validation (equal), visualization (equal), writing – original draft (equal), writing – review and editing (equal). **Heng‐Fang Wang:** conceptualization (equal), visualization (equal), writing – original draft (equal), writing – review and editing (equal). **Kai‐Hui Li:** data curation (equal), resources (equal), software (equal), supervision (equal), validation (equal). **Yan‐Yan Liu:** data curation (equal), formal analysis (equal), funding acquisition (equal), investigation (equal), project administration (equal), resources (equal), validation (equal). **Yan‐Ming Gong:** conceptualization (equal), data curation (equal), formal analysis (equal), methodology (equal), software (equal), supervision (equal), validation (equal), writing – original draft (equal), writing – review and editing (equal).

## Conflicts of Interest

The authors declare no conflicts of interest.

## Supporting information


Appendix S1.

## Data Availability

Data from this study are available and can be accessed at the public data repository Dryad. https://doi.org/10.5061/dryad.9s4mw6mss.
