# Supplementary material for: Opposing Effects of Plant Invasion on the Stability of Aboveground and Belowground Net Primary Productivity in an Alpine Grassland
Source: Ecol Evol. 2025 Jul 14;15(7):e71730. doi: 10.1002/ece3.71730 (PMC12256773; doi:10.1002/ece3.71730)
Supplement: Supplementary file 1 — Appendix S1. [file ECE3-15-e71730-s001.docx]

**Opposing Effects of Plant Invasion on the Stability of Above- and Belowground Net Primary Productivity in an Alpine Grassland**

**Appendix：**

**
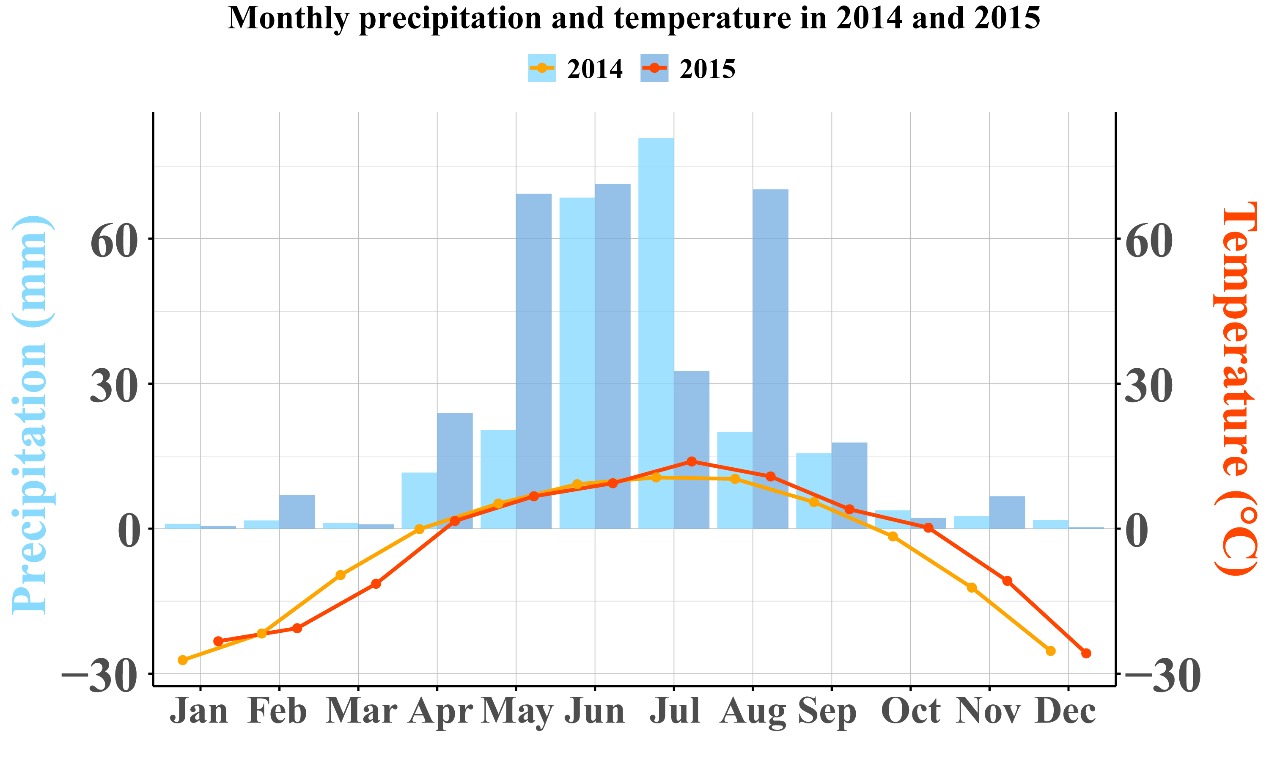
**

Figure S1. Monthly precipitation and temperature in 2014 and 2015.


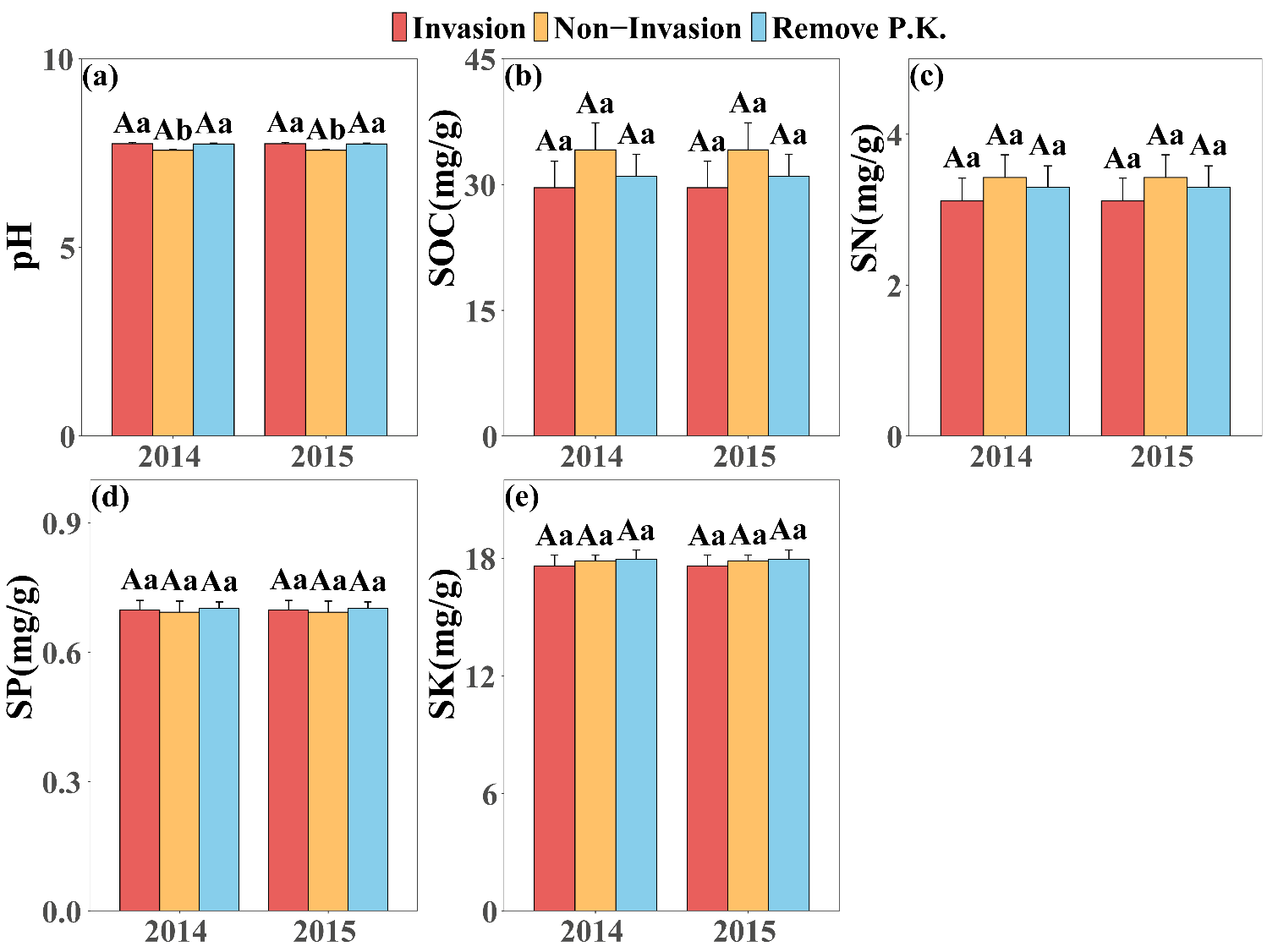


Figure S2. Physical and chemical properties of soil in 2014 and 2015.

Table S1 The classification information of plant species, families and genera, and the division of plant functional groups in the sample subplots

| plot | number | Family | Genus | Species | Plant functional group |
| --- | --- | --- | --- | --- | --- |
| 1 | 1 | *Plantaginaceae* | *Plantago* | *Plantago asiatica* | forbs |
| 1 | 2 | *Rosaceae* | *Potentilla* | *Potentilla multifida* | forbs |
| 1 | 3 | *Rosaceae* | *Argentina* | *Argentina anserina* | forbs |
| 1 | 4 | *Rosaceae* | *Sibbaldianthe* | *Sibbaldianthe bifurca* | forbs |
| 1 | 5 | *Asteraceae* | *Erigeron* | *Erigeron acris* | forbs |
| 1 | 6 | *Fabaceae* | *Astragalus* | *Astragalus membranaceus* | legumes |
| 1 | 7 | *Asteraceae* | *Leontopodium* | *Leontopodium leontopodioides* | forbs |
| 1 | 8 | *Fabaceae* | *Oxytropis* | *Oxytropis lapponica* | legumes |
| 1 | 9 | *Gentianaceae* | *Gentianella* | *Gentianella turkestanorum* | forbs |
| 1 | 10 | *Asteraceae* | *Cirsium* | *Cirsium esculentum* | forbs |
| 1 | 11 | *Gentianaceae* | *Gentiana* | *Gentiana scabra* | forbs |
| 1 | 12 | *Ranunculaceae* | *Ranunculus* | *Ranunculus japonicus* | forbs |
| 1 | 13 | *Poaceae* | *Elymus* | *Elymus dahuricus* | grass |
| 1 | 14 | *Asteraceae* | *Taraxacum* | *Taraxacum mongolicum* | forbs |
| 1 | 15 | *Poaceae* | *Koeleria* | *Koeleria macrantha* | grass |
| 1 | 16 | *Cyperaceae* | *Carex* | *Aegopodium* | forbs |
| 1 | 17 | *Poaceae* | *Festuca* | *Festuca kryloviana* | grass |
| 1 | 18 | *Poaceae* | *Stipa* | *Stipa purpurea* | grass |
| 2 | 1 | *Gramineae* | *Agropyron* | *Agropyron cristatum* | grass |
| 2 | 2 | *Plantaginaceae* | *Plantago* | *Plantago asiatica* | forbs |
| 2 | 3 | *Rosaceae* | *Potentilla* | *Potentilla multifida* | forbs |
| 2 | 4 | *Rosaceae* | *Argentina* | *Argentina anserina* | forbs |
| 2 | 5 | *Rosaceae* | *Sibbaldianthe* | *Sibbaldianthe bifurca* | forbs |
| 2 | 6 | *Asteraceae* | *Erigeron* | *Erigeron acris* | forbs |
| 2 | 7 | *Boraginaceae* | *Lappula* | *Lappula myosotis* | forbs |
| 2 | 8 | *Fabaceae* | *Astragalus* | *Astragalus membranaceus* | legumes |
| 2 | 9 | *Asteraceae* | *Leontopodium* | *Leontopodium leontopodioides* | forbs |
| 2 | 10 | *Fabaceae* | *Oxytropis* | *Oxytropis lapponica* | legumes |
| 2 | 11 | *Gentianaceae* | *Gentianella* | *Gentianella turkestanorum* | forbs |
| 2 | 12 | *Asteraceae* | *Cirsium* | *Cirsium esculentum* | forbs |
| 2 | 13 | *Gentianaceae* | *Gentiana* | *Gentiana scabra* | forbs |
| 2 | 14 | *Ranunculaceae* | *Ranunculus* | *Ranunculus japonicus* | forbs |
| 2 | 15 | *Poaceae* | *Elymus* | *Elymus dahuricus* | grass |
| 2 | 16 | *Asteraceae* | *Taraxacum* | *Taraxacum mongolicum* | forbs |
| 2 | 17 | *Poaceae* | *Koeleria* | *Koeleria macrantha* | grass |
| 2 | 18 | *Gentianaceae* | *Gentiana* | *Gentiana macrophylla* | forbs |
| 2 | 19 | *Apiaceae* | *Aegopodium* | *Aegopodium alpestre* | forbs |
| 2 | 20 | *Cyperaceae* | *Carex* | *Aegopodium* | forbs |
| 2 | 21 | *Poaceae* | *Festuca* | *Festuca kryloviana* | grass |
| 2 | 22 | *Poaceae* | *Stipa* | *Stipa purpurea* | grass |
| 3 | 1 | *Gramineae* | *Agropyron* | *Agropyron cristatum* | grass |
| 3 | 2 | *Plantaginaceae* | *Plantago* | *Plantago asiatica* | forbs |
| 3 | 3 | *Plantaginaceae* | *Plantago* | *Plantago asiatica* | forbs |
| 3 | 4 | *Rosaceae* | *Potentilla* | *Potentilla multifida* | forbs |
| 3 | 5 | *Rosaceae* | *Argentina* | *Argentina anserina* | forbs |
| 3 | 6 | *Rosaceae* | *Sibbaldianthe* | *Sibbaldianthe bifurca* | forbs |
| 3 | 7 | *Asteraceae* | *Erigeron* | *Erigeron acris* | forbs |
| 3 | 8 | *Boraginaceae* | *Lappula* | *Lappula myosotis* | forbs |
| 3 | 9 | *Fabaceae* | *Astragalus* | *Astragalus membranaceus* | legumes |
| 3 | 10 | *Asteraceae* | *Leontopodium* | *Leontopodium leontopodioides* | forbs |
| 3 | 11 | *Fabaceae* | *Oxytropis* | *Oxytropis lapponica* | legumes |
| 3 | 12 | *Gentianaceae* | *Gentianella* | *Gentianella turkestanorum* | forbs |
| 3 | 13 | *Asteraceae* | *Cirsium* | *Cirsium esculentum* | forbs |
| 3 | 14 | *Gentianaceae* | *Gentiana* | *Gentiana scabra* | forbs |
| 3 | 15 | *Ranunculaceae* | *Ranunculus* | *Ranunculus japonicus* | forbs |
| 3 | 16 | *Poaceae* | *Elymus* | *Elymus dahuricus* | grass |
| 3 | 17 | *Asteraceae* | *Taraxacum* | *Taraxacum mongolicum* | forbs |
| 3 | 18 | *Poaceae* | *Koeleria* | *Koeleria macrantha* | grass |
| 3 | 19 | *Cyperaceae* | *Carex* | *Aegopodium* | forbs |
| 3 | 20 | *Poaceae* | *Festuca* | *Festuca kryloviana* | grass |
| 3 | 21 | *Poaceae* | *Stipa* | *Stipa purpurea* | grass |
| 4 | 1 | *Gramineae* | *Agropyron* | *Agropyron cristatum* | grass |
| 4 | 2 | *Plantaginaceae* | *Plantago* | *Plantago asiatica* | forbs |
| 4 | 3 | *Rosaceae* | *Potentilla* | *Potentilla multifida* | forbs |
| 4 | 4 | *Rosaceae* | *Argentina* | *Argentina anserina* | forbs |
| 4 | 5 | *Rosaceae* | *Sibbaldianthe* | *Sibbaldianthe bifurca* | forbs |
| 4 | 6 | *Asteraceae* | *Erigeron* | *Erigeron acris* | forbs |
| 4 | 7 | *Boraginaceae* | *Lappula* | *Lappula myosotis* | forbs |
| 4 | 8 | *Fabaceae* | *Astragalus* | *Astragalus membranaceus* | legumes |
| 4 | 9 | *Asteraceae* | *Leontopodium* | *Leontopodium leontopodioides* | forbs |
| 4 | 10 | *Fabaceae* | *Oxytropis* | *Oxytropis lapponica* | legumes |
| 4 | 11 | *Gentianaceae* | *Gentianella* | *Gentianella turkestanorum* | forbs |
| 4 | 12 | *Asteraceae* | *Cirsium* | *Cirsium esculentum* | forbs |
| 4 | 13 | *Gentianaceae* | *Gentiana* | *Gentiana scabra* | forbs |
| 4 | 14 | *Ranunculaceae* | *Ranunculus* | *Ranunculus japonicus* | forbs |
| 4 | 15 | *Poaceae* | *Elymus* | *Elymus dahuricus* | grass |
| 4 | 16 | *Asteraceae* | *Taraxacum* | *Taraxacum mongolicum* | forbs |
| 4 | 17 | *Poaceae* | *Koeleria* | *Koeleria macrantha* | grass |
| 4 | 18 | *Gentianaceae* | *Gentiana* | *Gentiana macrophylla* | forbs |
| 4 | 19 | *Cyperaceae* | *Carex* | *Aegopodium* | forbs |
| 4 | 20 | *Poaceae* | *Festuca* | *Festuca kryloviana* | grass |
| 4 | 21 | *Poaceae* | *Stipa* | *Stipa purpurea* | grass |
| 5 | 1 | *Gramineae* | *Agropyron* | *Agropyron cristatum* | grass |
| 5 | 2 | *Plantaginaceae* | *Plantago* | *Plantago asiatica* | forbs |
| 5 | 3 | *Rosaceae* | *Potentilla* | *Potentilla multifida* | forbs |
| 5 | 4 | *Rosaceae* | *Argentina* | *Argentina anserina* | forbs |
| 5 | 5 | *Rosaceae* | *Sibbaldianthe* | *Sibbaldianthe bifurca* | forbs |
| 5 | 6 | *Asteraceae* | *Erigeron* | *Erigeron acris* | forbs |
| 5 | 7 | *Asteraceae* | *Chondrilla* | *Chondrilla piptocoma* | forbs |
| 5 | 8 | *Boraginaceae* | *Lappula* | *Lappula myosotis* | forbs |
| 5 | 9 | *Fabaceae* | *Astragalus* | *Astragalus membranaceus* | legumes |
| 5 | 10 | *Asteraceae* | *Leontopodium* | *Leontopodium leontopodioides* | forbs |
| 5 | 11 | *Fabaceae* | *Oxytropis* | *Oxytropis lapponica* | legumes |
| 5 | 12 | *Gentianaceae* | *Gentianella* | *Gentianella turkestanorum* | forbs |
| 5 | 13 | *Asteraceae* | *Cirsium* | *Cirsium esculentum* | forbs |
| 5 | 14 | *Gentianaceae* | *Gentiana* | *Gentiana scabra* | forbs |
| 5 | 15 | *Ranunculaceae* | *Ranunculus* | *Ranunculus japonicus* | forbs |
| 5 | 16 | *Poaceae* | *Elymus* | *Elymus dahuricus* | grass |
| 5 | 17 | *Asteraceae* | *Taraxacum* | *Taraxacum mongolicum* | forbs |
| 5 | 18 | *Poaceae* | *Koeleria* | *Koeleria macrantha* | grass |
| 5 | 19 | *Gentianaceae* | *Gentiana* | *Gentiana macrophylla* | forbs |
| 5 | 20 | *Apiaceae* | *Aegopodium* | *Aegopodium alpestre* | forbs |
| 5 | 21 | *Cyperaceae* | *Carex* | *Aegopodium* | forbs |
| 5 | 22 | *Poaceae* | *Festuca* | *Festuca kryloviana* | grass |
| 5 | 23 | *Poaceae* | *Stipa* | *Stipa purpurea* | grass |
